# How do medical students in their clinical years perceive basic sciences courses at King Saud University?

**DOI:** 10.4103/0256-4947.75780

**Published:** 2011

**Authors:** Awatif Alam

**Affiliations:** From the Department of Family and Community Medicine, College of Medicine, King Saud University and KSU Research Chair of Epidemiology and Public Health, Riyadh, Saudi Arabia

## Abstract

**BACKGROUND AND OBJECTIVES:**

The inclusion of detailed basic science courses in medical school curricula has been a concern of students. The main objective of this study was to explore the attitudes of medical students towards basic sciences courses taught to them in the preclinical years and the applicability of these courses to current clinical practice.

**DESIGN AND SETTING:**

A cross-sectional survey was conducted during 2008-2009 among medical students in their clinical years at King Saud University, Riyadh, Saudi Arabia.

**METHODS:**

Thirty percent of all students (n=314) were randomly selected to receive a questionnaire designed to evaluate their opinions about course load, ability to recall information, value of practical sessions, availability of references and course guidelines, and the applicability of individual courses to clinical practice.

**RESULTS:**

Students identified anatomy and pathology as the courses most overloaded with content (76% and 70%, respectively). Half of the students felt they retained the most knowledge of physiology (50%), while less than a quarter of students (19%) felt they retained the most information from biochemistry coursework. The role of practical sessions in facilitating theoretical understanding was more evident in anatomy (69%). Physiology was perceived as the subject with the highest applicability to clinical practice (66%), while pathology (29%) was identified as the subject with the least practical application. Students became increasingly negative in their opinions about basic science courses as they progressed through their medical education.

**CONCLUSION:**

Current attitudes of medical students towards their basic science courses indicate a need to reform the curricula so as to maximize the benefit of these courses.

There is growing concern among medical educators that traditional programs of teaching medical students have not provided better outcomes of learning.^1^,^2^ For instance, the first 2 years of medical study at King Saud University (KSU) are devoted to a basic sciences program, which does not include patient-based teaching. Little emphasis is placed on how the knowledge or skills will be used in courses taught at a later stage. Under such circumstances, students mainly concentrate on ways to gain marks, rather than focusing on how the information could be applied in clinical settings. Generally, students see learning as a process whereby knowledge is provided to them by the teacher, and they view the curriculum as an aggregate of separate subjects (e.g., pathology, anatomy, etc.), rather than a single general topic (e.g., medicine). In 2008, Custers wrote that the “longevity of science knowledge learned in medical school has been a source of concern, probably for as long as this knowledge is [sic] included in the curriculum.”^3^ Generally, medical students in conventional programs use previously learned basic sciences knowledge to build their clinical knowledge. Many senior undergraduate students have indicated that they did not remember much from their basic science medical courses and that the content of those courses did not seem relevant to their later clinical employment or studies.^4^,^5^ Evidence collected from several medical schools called for following explicit objectives for each course to achieve recommended outcomes for graduation.^6^

It is believed that student achievements and future practice are affected by their attitudes towards basic sciences courses. The main objective of this study was to explore how medical students in their clinical years feel about the basic sciences courses taught to them in the preclinical years, and to assess how students rate the applicability of these courses to current clinical training.

## METHODS

A cross-sectional descriptive survey was conducted during the 2008-2009 academic year among medical students enrolled in the third, fourth and fifth years of their clinical program at King Saud University in Riyadh, Saudi Arabia, which adopts conventional basic sciences curricula. A total of 940 students were enrolled, cumulatively, in the third, fourth and fifth years. Thirty percent (314) of these students were randomly selected to be a part of the survey. The survey was approved by the college research committee, and verbal consent was obtained from students prior to their participation in the study.

Data were collected using an anonymous, pre-designed, self-administered questionnaire. Pre-testing was conducted using a sample of 30 students, who were asked to comment on the questionnaire’s content, consistency, clarity and appearance; necessary modifications were adopted as recommended. The questionnaire collected information on students’ academic year and their perceptions of the following characteristics of their basic sciences courses (anatomy, pathology, biochemistry, microbiology and physiology): overload, recall of information, integration (between theoretical and practical sessions), availability of references lists, course specifications, and applicability to clinical practice. All opinions were rated using the 5-point Likert scale, which ranges from “strongly disagree” to “strongly agree.” Although all opinion levels were initially analyzed, percentages of those who responded as either “strongly agree” or “agree” were merged into a single category, and tables were prepared so as to present only positive responses. Data were arranged as numbers and percentages. All responses were stratified by academic year. Chi-square tests were performed on categorical variables, and significance was defined as *P*<.05.

## RESULTS

Of the 314 survey participants, 111 (35.4%) were third-year students, 98 (31.2%) were fourth-year students and 105 (33.4%) were fifth-year medical students. The study group included students of both genders (69% males, 31% females), which is proportional to the gender ratio of students entering college. The majority of medical students perceived anatomy and pathology as the courses most overloaded with content (76% and 70%, respectively) (**Table 1**). Overall, students did not feel that they could recall information learned in their basic sciences courses. Fifty percent of the students indicated that they retained the most knowledge from physiology coursework, while 19% of the students indicated that they retained the most knowledge from biochemistry coursework. The contribution of practical sessions in facilitating theoretical understanding was the highest for anatomy (69% of students), and the least percentage (21%) of students felt the same for biochemistry. Students in each academic year ranked pathology and microbiology the lowest in terms of providing lists of references and course specifications. Physiology was perceived as the subject with the highest applicability to clinical practice (66% of students), followed by biochemistry (61%) and anatomy (50%); pathology ranked lowest (29%).

**Table 1:** Attitudes* of third-, fourth- and fifth-year medical students towards selected features of basic science courses.

Features	Anatomy				Pathology				Biochemistry				Microbiology				Physiology			
	3rd n (%)	4th n (%)	5th n (%)	Total n (%)	3rd n (%)	4th n (%)	5th n (%)	Total n (%)	3rd n (%)	4th n (%)	5th n (%)	Total n (%)	3rd n (%)	4th n (%)	5th n (%)	Total n (%)	3rd n (%)	4th n (%)	5th n (%)	Total n (%)
Overloaded courses	87 (78)	74 (76)	78 (74)	239 (76)	85 (77)	65 (66)	71 (68)	221 (70)	53 (48)	57 (58)	43 (41)	153 (49)	50 (45)	45 (46)	42 (40)	137 (44)	45 (41)	49 (50)	44 (42)	138 (44)
Knowledge recall	23 (21)	22 (22)	21 (20)	66 (21)	25 (23)	18 (18)	21 (20)	64 (20)	24 (22)	19 (19)	15 (14)	58 (19)	24 (22)	19 (19)	20 (19)	63 (20)	61 (55)	51 (52)	44 (42)	156 (50)
Practical integration	79 (71)	61 (62)	75 (71)	215 (69)	39 (35)	38 (39)	33 (31)	110 (35)	21 (19)	29 (30)	17 (16)	67 (21)	50 (45)	37 (38)	38 (36)	125 (40)	50 (45)	51 (52)	41 (39)	142 (45)
Available references	87 (78)	80 (82)	81 (77)	248 (79)	49 (44)	46 (47)	46 (44)	141 (45)	96 (87)	88 (90)	93 (89)	277 (88)	30 (27)	37 (38)	18^b^ (17)	85 (27)	63 (57)	62 (63)	65 (62)	190 (61)
Course specifications	79 (71)	57 (58)	59 (56)	159 (62)	56 (51)	43 (44)	39 (37)	138 (44)	79 (71)	68 (69)	63 (60)	210 (67)	46 (41)	45 (46)	40 (38)	131 (42)	81 (73)	67 (68)	62 (59)	210 (67)
Applicability to clinical practice	69^a^ (62)	37 (38)	52 (50)	158 (50)	40 (36)	27 (28)	24 (23)	91 (29)	81 (73)	55 (56)	56 (53)	192 (61)	50 (45)	36 (37)	34 (32)	120 (38)	85^c^ (77)	63 (64)	59 (56)	207 (66)

Third-year medical students: n=111; fourth-year medical students: n=98; fifth-year medical students: n=105.

* Students who agreed about given feature.

a *P*= .011,

^b^*P*= .025,

c *P*=.031 comparing across academic years.

As students progressed from their third year through the fifth year, they became increasingly negative about their educational experience. For instance, more students from the higher classes felt that basic sciences curricula were highly overloaded, that they could recall less information, and that curricula were less applicable to clinical practice (*P*=.011 for anatomy, *P*=.025 for microbiology, *P*=.031 for physiology).

## DISCUSSION

Traditional medical education programs have been criticized for a variety of reasons, including overcrowding, overemphasis on some subjects, presence of relatively nonrelevant topics, a dissociation between basic and clinical sciences, and repetition of lectures.^6^ To diminish these problems, some have suggested altering the curriculum to ensure vertical integration, by initially teaching only clinically-oriented basic science subjects and then providing additional learning experiences in these subjects during the clinical years.^7^–^9^ The results showed a paradoxical association between students’ perception of receiving overloaded courses, being able to recall information, and receiving information that was applicable to clinical practice.

One solution to content overload may be establishing a core curriculum with additional options or special study modules.^10^ This method would ensure that students acquire the knowledge, skills and attitudes required for maintaining educational standards, while also allowing them to take more responsibility for their own learning.^10^

Both within academic years and across academic years, students reported an inability to recall information for almost all basic sciences courses. This may be related to volume overload, lack of knowledge application, and the teaching method. Indeed, previous research has shown that student knowledge retention decreases over time and that these decreases occur at different rates for different courses.^11^ While some authors considered retained memory and quality of learning as central for medical education, others argued that knowledge which cannot be of use becomes inert and inaccessible.^12^–^14^

Paradoxically, students ranked biochemistry lowest in terms of integration between theory and practice, as well as in terms of recall of information, but did not give it a correspondingly low rank in terms of its applicability to clinical practice. This may have been caused by the complex interplay among factors, which necessitates understanding the cognitive processes regulating how students think and learn.

At the turn of the century, Brownell reported that several medical schools in North America had introduced innovations in the teaching of basic sciences in order to integrate basic and clinical science content and to promote active student learning.^6^ Similarly, profound changes have been brought about in medical education at KSU during the 2009-2010 academic year. With the adoption of the integrated system in the first year, there has been a shift from faculty-centered to student-centered learning, with an increase in small-group discussions, tutorials and students’ use of the library for self-directed learning. In general, this has greatly reduced reliance on lectures for the transfer of knowledge. Similar changes have been made at many medical schools, as exemplified by the recent statements of the Association of American Medical Colleges, in 2009, which encouraged innovation in the design of premedical and medical curricula by focusing on competencies rather than on specific courses taken or disciplines studied.^15^

In conclusion, current attitudes of students towards basic science courses across the clinical study years are alarming and warrant further consideration and analysis. At institutions such as KSU, future studies will be needed to compare the competencies of students enrolled in the courses with integrated medical curricula versus those of students studying the conventional medical curricula, as well as to evaluate student perception of medical education. The overall goals of medical education include the attainment of knowledge, skills, attitudes and values required to perform professional medical tasks competently and safely; it is necessary to better integrate clinical experiences and basic sciences courses in order to achieve these goals.
